# Finding the right connection: what makes a successful decision support system?

**DOI:** 10.1002/fes3.123

**Published:** 2017-12-25

**Authors:** David C. Rose, Toby J. A. Bruce

**Affiliations:** ^1^ School of Environmental Sciences University of East Anglia Norwich Research Park Norwich UK; ^2^ School of Life Sciences Keele University Keele, Staffordshire UK

## Abstract

Farmers require evidence‐based guidance to make optimal decisions, enabling them to reduce costs by increasing the efficiency of input use. We discuss how decision support systems could be improved and made more useful for the farmer.

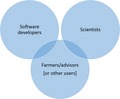

There is growing recognition that the agricultural industry is undergoing a period of transformation to become a more information‐intensive enterprise (Bruce, 2016; Wolfert, Ge, Verdouw, & Bogaardt, 2017). There is much talk of how “big data” will help farmers and how an “Internet of things” will allow optimization of inputs such as water, fertilizer, and pesticide through the use of precision sensors (Wolfert et al., 2017). Furthermore, research is considering how decision support systems might be able to present information in a useable format for on‐farm decision making (Lindblom, Lundström, Ljung, & Jonsson, 2017; Rose et al., 2016). Such shifts toward an increasingly technical mode of agriculture comes at a time when farmers are facing a number of challenges, such as rising input costs, and stagnating commodity prices. Crop protection in particular is becoming increasingly challenging as pests, weeds, and diseases evolve resistance to pesticides, and legislative restrictions reduce the options available (Bruce, 2016). To address these threats, farmers require evidence‐based guidance to make optimal decisions.

As we enter an ever‐more sophisticated information age, there is a need to bring science and farming communities together to turn information into relevant, actionable farming knowledge (Allen, Cruz, & Warburton, 2017; Bruce, 2016; Oliver, Bartie, Louise Heathwaite, Pschetz, & Quillam, 2017; Rose et al., 2017). As part of an advisory network, which includes trusted advisers and support networks, there is enormous potential for web‐based knowledge exchange to facilitate two‐way flow of information to and from farms, and to share information about “what works” (Bruce, 2016).

To ensure that knowledge is useable and actionable, information should be collated into a userfriendly format, and decision support systems (DSS) are one suggested solution to deliver it to practitioners (Dicks, Walsh, & Sutherland, 2014). These are usually software systems, which are increasingly app‐based, and lead users through evidence‐based decision stages toward a final decision (Dicks et al., 2014). One benefit of DSS could be to enable smarter use of inputs, thereby having positive implications for finances and the environment. Indeed, this could help to address one of the key global 21st century challenges, which is to maximize agricultural production while minimizing use of resources such as land, water, and energy to meet rising demand for produce (Bruce, 2016). However, if decision support tools are going to be used, and to make a difference in practice, we argue that better‐designed DSS are required that are fit for purpose, and are relevant to local needs (Wood et al., 2014).

To expedite improvements, a step‐change is needed within the design of agricultural DSS. Design teams are based in a variety of places, including universities, commercial software companies, and elsewhere in the agricultural industry. Although there are examples of successful DSS, which are used and well liked in practice, many suffer from similar design flaws, which restrict uptake (Rose et al., 2016). For example, Rose et al. (2017) present the example of “Tool X” (anonymized), a fertilizer application system designed to address flaws in an existing system. These flaws included lack of reliability, lack of flexibility on units of measurements, and the difficulty of undoing mistakes when a farmer inputted data. Lack of system uptake by farmers has been noted for several decades (e.g., Parker & Sinclair, 2001), but recent work has again highlighted the same salient points (e.g., Rose et al., 2016), renewing calls for a change to design cultures.

This recent work has still found that DSS are sometimes not easy to use, answer the wrong questions, fail to fit the workflow or decision habits of farmers, are too costly, lack a clear purpose, are poorly marketed, and lack any clear long‐term maintenance plan (among other factors—see Rose et al., 2016). Yet, a series of good, practical suggestions have been made to improve the user‐centered design of DSS in order that farmer and adviser needs can be taken into account (Rose et al., 2017). These include a six‐step process of user‐centered design: (1) identifying the user and their workflow (e.g., farmer or adviser?), (2) asking if, and how, the user would benefit, (3) investigating whether rural infrastructure is in place for the tool to be used, (4) testing, with actual users instead of colleagues, whether the system is easy to use, (5) adopting a good delivery plan, considering peer‐to‐peer networking and trusted advisory networks, and (6) thinking about how the system will be maintained after release, otherwise it will quickly become obsolete (see Rose et al., 2017). Where farmers and advisers have been included in the design from the outset, DSS have been better targeted, much easier to use, and provided enhanced benefits (Allen et al., 2017; Oliver et al., 2017).

We encourage developers of DSS to take heed of the good advice already in the literature; first, to consult users from the outset; second, to include team members with specialist user‐oriented software knowledge (Lindblom et al., 2017); and third, to pursue impact as a measure of success beyond academic publication. These three stages require a culture shift for those designing systems, one which acknowledges that farmers must be consulted from the outset, and further that scientific sophistication should never trump the needs and views of those who are doing the farming.

## CONFLICT OF INTEREST

None declared.
